# An Essay on the Mucous Membranes

**Published:** 1845-12

**Authors:** John Overton

**Affiliations:** Nashville, Tenn.


Art. II.—*An Essay on the Mucous Membranes.* Read before the Medical Society of Tennessee, in May, 1845. By John Overton, M.D., of Nashville, Tenn.

At the last meeting of the Society I promised to submit to the present session a dissertation on some subject properly included within the domain of medical investigation. In the redemption of that promise, I have chosen for the subject of the few observations which follow, the *mucous membranes* of the human body, as they are constituted and distributed in its ordinary construction.

The membranes of the human body considered collectively, and as constituting a system of organs distinguished by the character and strong analogy of their organization and function, may justly be esteemed a subject of pre-eminent importance to the medical profession; and consequently entitled to receive a greatly augmented portion of professional research and reflection.

It is a subject of import, much too broad to be reduced within the limits of an essay like the present, and I have therefore deemed it proper to refer merely to the ample and interesting field which it opens to medical inquiry, abstaining from any examination of its important and multitudinous details, which would be incompatible with the single object proposed by this paper; of which the almost exclusive design is to engage, if I may, in some greater degree, the professional attention of my State to the study of the mucous membranes in their healthy and pathological conditions.

The obscurity of situation, and the minuteness of their physical structure, together with the less prominence of their functions in the human economy, have operated chiefly to deprive these organs of the early and thorough examination which, in the progress of medical science, the profession has given to other organs and functions of greater prominence and palpability of anatomical and physiological character, but not greater importance in the animal economy to the perfect and healthy execution of its various and wonderful functions.

It is from these causes, perhaps, more than from any other, that for a long succession of ages, anatomy, even, had achieved no exact ideas in relation to these membranes. Other portions of the human body had been exactly demonstrated, and their uses and functions clearly ascertained, whilst an impenetrable cloud still veiled from professional comprehension the structure and functions of this highly important class of organs in the animal system. None had imagined that by their assemblage they constituted a distinct and important system of organs; their varieties even were imperfectly or not at all recognized and designated; and they were hence, often, in anatomical description, confounded with other tissues of dissimilar character. It is true that many of the individual membranes, occupying special localities of the body were early noticed and anatomically described; but any successful attempt to arrange the different membranes of the body into a distinct system of organs, to fix beyond the reach of future question their physical structure, functions, and various pathological states and relations, both healthy and diseased, was denied to the earlier cultivators of our art; and was only to be consummated by the more technical processes, and recondite reasearches of modern investigators.

Until within the last twenty-five or thirty years, the knowledge of the profession in relation to the membranes of the body, was restricted to a few isolated facts, which contributed nothing, or but little, towards any thing like valuable results for the improvement of practical medicine, and could be looked upon chiefly, if not exclusively, as evidences only of anatomical curiosity, fruitless of any valuable influence upon the science of healing diseases.

Upon this chaos, which had lasted for so many ages, in reference to this greatly important subject of medical research, the labors of the profession had conferred neither system nor order. Everything remained, of what little was known, in wide dispersion over the entire field of medical inquiry, without system or profitable consequences to the profession, when the observation fortunately occurred to Mr. Pinel, that the diseases of the different membranes of the body were connected with each other by close and intimate relations; and that these relations were in subordination to the greater or less identity of the anatomical structure of the different membranes in which they obtained. This parent observation of a single man, distinguished for the strength and perspicuity of his intellect, and animated by a laudable zeal for the advancement of medical science, has given birth to doctrines and practice in medicine, of whose extent and importance it is difficult if not impossible to form even an adequate conception.

An idea destined so palpably to exercise an important influence upon practical medicine, could not be overlooked, or fail to attract the early and serious attention which it so justly merited from the profession. Mr. Pinel, as before intimated, had established upon a basis of well conducted observation, that the different membranes of the body were grouped by nature into distinct classes and orders; and that the diseases to which these different classes and orders were liable, were marked by identity of character and features, in accordance with the class or order of membranes in which they occurred—that phrenitis, pleurisy, and peritonitis, for example, are inflammations of the same class and order of membranes, and that their diseases are characterised by phenomena which are common to them all, and which demand for their cure, correspondently identical or similar therapeutic treatment.

These are, however, inflammations of serous membranes, occupying, it is true, distinct and widely different portions in the system, but notwithstanding this distinction, identical in their anatomical structure, functions, pathological liabilities, and thearapeutical relations. The same, or similar relations, were established between other classes of membranes, as coryza, pulmonary catarrh, leucorrhœa, and some diarrhœas, all of which being inflammations of the mucous membranes, were distinguished by common features, giving ample indication of the identity of organization of the parts in which they were located, and of the generic and similar therapeutic treatment, best calculated to insure their restoration to health when in a morbid state.

Pathological anatomy and therapeutical experience in subsequent practice added their reliable testimony to those inceptive opinions of Mr. Pinel—the professional mind stimulated by these fortunate views and observations of their illustrious author, was suddenly roused from its long stupor and neglect of this highly interesting, and till then unbroken field of medical research; and the study and investigation of the membranes soon became a subject of popular and profitable pursuit to many eminent members of the medical profession.

It constituted an important and imperishable epoch in the progress of medical science, and has contributed to an extent not easily to be sufficiently appreciated, towards the establishment of the present improved state and character of medical practice throughout the entire range of its exercise. It may be affirmed, it is believed, with but little hazard of successful contradiction, that no one discovery, save that of the circulation of the blood by Harvey, has done more towards the cure of diseases, and mitigation of human suffering, than this brilliant and incalculably valuable observation, first made and subsequently established to the imperishable renown of its illustrious author. Harvey, Pinel, and Bichat were congenial and kindred spirits, and have each left inscribed upon the pages of medical history, monuments of intellectual triumphs more durable and brilliant in their material, than Parian marble, or the celebrated brass of ancient Corinth—monuments which defy the power of corrosive time to deface or destroy, and which naught but the overwhelming might of ignorance and barbarism will ever again conceal from the gratitude and admiration of the world.

They may, it is admitted, be obscured and fall into comparative neglect for a time, under the influence of other popular and apparently contradictory doctrines and facts in medicine, but their permanence and value are established upon the solid rock of exact observation and sound and logical induction, and cannot hence be overborne by the power of time or scrutiny. They will stand in all future time as fixed points, as beacon lights, upon the ocean of medical science, to direct the course of the puzzled and benighted practitioner into the haven of safe and successful medication.

Too much praise cannot be ascribed to Mr. Pinel and to his contemporaries and immediately succeeding co-laborers in this very important department of medical research.

But the special object of this paper being to ask from the profession some greater attention to the mucous membranes, it may be esteemed irrelavent, or an improper digression to give further extension to remarks on the membranes in general. Enough has been said, it is thought, to indicate their vast importance to the medical practitioner, and as necessarily and intimately connected in structure and function, with the individual class of membranes which is the special object of this essay.

Nor will it comport with the distinction imposed by the present occasion, to enter upon any detailed anatomical description of the mucous membranes, in connection with their physiological functions.

Such an examination would involve a task greatly too extensive and important in its character, to be executed with profit upon the present occasion, but which merits to receive the most careful and profound attention of him who is engaged, or designs to be occupied, with the treatment of human diseases.

Leaving then untouched the anatomy and physiology of the mucous membranes, as objects too extensive and important to be condensed within the narrow scope of this paper, I deem it proper to confine myself to some of the more prominent characters of vitality, by which the mucous membranes are distinguished in their normal and diseased states; and to indicate merely the localities upon which these membranes are chiefly distributed in the human body.

The mucous membranes of the body are chiefly occupied by the investment of the internal surface of the intestinal canal, including the entire tube, however diversified in its structure, functions and form, which reaches from the mouth to the anus; the internal surface of the trachea and bronchia of the lungs; the internal surface of the urinary bladder and urethra in man, and the vagina and uterus in woman; and the nose and nasal fossæ; together with the cutaneous tissue which covers the external surface of the body, and which on many accounts may be considered a mucous membrane, modified in aspect and function by diversity of location, uses, and the peculiar physical agencies with which it is perpetually in contact, and which are aptly constituted to excite its activity.

From the strong analogies existing between the mucous membrane and the skin, the former has been, as it is thought, aptly denominated the internal skin, as a distinction which may profitably indicate its similarity of anatomical structure and physiological function—the mucous membranes performing for the hollow organs, upon whose internal surface they are distributed, an office closely analogous to that of the cutaneous tissue upon the external surface of the body. The intimate relations existing between these two surfaces, has been noticed and admitted by most accurate observers of the functions of human life, and merits a full share of the attention of every medical practitioner. It is a subject for investigation, which cannot be easily over estimated, as a guide to the therapeutical treatment of many important maladies. It should be diligently considered and carefully respected, in every process intended for the preservation of health or the modification of disease.

The intimate and important relation of the mucous membranes with the cutaneous tissue is most palpable, and best understood, as it is exhibited in exanthematous diseases, in measles and scarlatina, and other similar affections.

In these, it has been clearly demonstrated, by the labors of those engaged in the cultivation of pathological anatomy, that the cutaneous affection, which until recently, has been considered as primary and essential, is only symptomatic of the real malady, which consists in an inflammation, or at least in violent irritation, of the mucous membrane of the stomach and bowels; and which in their progress excite into analogous morbid activity, the cutaneous tissue which is the seat of the visible eruption on the surface of the body. The disease is *internal* upon the mucous membrane, its visible effect, or symptom, or evidence of its existence, is *external*, upon the surface of the skin. But this eruption upon the skin, is symptomatic only of the real disease within, occupying the mucous membrane, and hence, in the medication of these diseases is entitled to receive an attention from the practitioner regulated and limited by a fixed estimate of its subordinate importance in the real character of the malady, of which it is merely an external and visible symptom.

Other sympathetic relations of a character less palpable, are known to exist also between the different mucous membranes of the body and the cutaneous tissue, by which it is invested; they are numerous and important in their influence, both in health and disease, but need not, it is thought, be enumerated in the present connection. Let it suffice to remark, as worthy of all attention, that the structure and functions of the mucous membranes, and those of the skin are related by analogies which are close and important, and which might well have intimated in the absence of other testimony, the many sympathies which exist necessarily between them, in their states both of health and disease.

Thus it appears from anatomical examination, that the external surface of the mucous membrane is, like the skin, covered by an epidermis of analogous anatomical structure, and designed to do an office in defending the nervous papillæ of the membrane from the too rude impressions of agents with which it may be brought in contact, remarkably similar to the functions of the epidermis on the external surface of the cutaneous tissue. This analogy in structure and function of the two tissues, is strikingly close; so much so as to have induced the belief of their probable identity—an idea which has drawn support of no feeble power, also from the similarity of the phenomena presented by both, in many of their morbid states, all tending to the conclusion, that the mucous membranes and the skin are identical in structure and functions, with the diversity only in both, which may be attributed to different location, and the diverse physical qualities of the objects with which they are usually in contact.

The resemblance of structure and function alluded to, may with sufficient accuracy, be likened to the almost identical structure and function of the bark of the stem or trunk, and that of the root, which obtains and is manifest to common observation in trees and other vegetables.

The bark upon the roots of trees and other vegetable bodies performs an office for the entire being, stem, branches, and organs of reproduction, very analogous, if not identical with the mucous membranes or internal skin in man and other animals. They both are designed to assimilate and unite to their organism, the heterogeneous elements of other substances with which they are placed in contact, and that too by a process similarly noiseless and unobtrusive in their modus operandi. The organic agent in both vegetables and animals—the root-bark in the one, and the mucous membranes in the other, are alike withdrawn from common inspection; and give evidence only of their actual condition by effects produced upon portions of their organism, often placed at a considerable distance from the organ whose activity has produced them. Thus we judge with much accuracy of the health of a horse, a man, or a tree, by the condition of the skin in the first instances, and of the bark or trunk-skin in the second.

But the condition of the skin in animals, and that of the trunk bark in vegetables, are both dependent upon and owe their existence, to the actual condition of mucous membranes in the one, and root-bark in the other; and observation has established the fact, that from a knowledge of the condition of the former, we may accurately deduce the actual condition of the latter; when the skin of animals is smooth, pliable, and soft, with a healthy transmission of insensible perspiration, we confidently infer that the assimilating organs, as the stomach and bowels, and the organs of respiration, are in a healthy condition. And in like manner, when the bark of vegetables is smooth, soft, and shining, we justly infer that the root is healthy and in the exercise of its healthy functions—functions which are exclusively performed by its cortical portion, without perhaps the slightest assistance from its central or ligneous portion, which serves only to give form and support to the assimilating organ, constituted by the root-bark. There is still another analogy between the relation which the root-bark in vegetables bears to the common bark of their stem and branches, and the relation of the mucous membrane or internal skin in animals with the cutaneous tissue, which forms the integument of the external surface of their bodies. If the roots of vegetables, and especially of trees, be elevated above the ground, leaving, however, a sufficiency of them in contact with it, to sustain the vitality of the individual which is subjected to the experiment, the portion of the root thus elevated and placed in constant contact with the atmosphere, loses in a short time, its primitive thin and delicate texture, with its loss of former function, and assumes the firmer and rougher texture of the common bark of the trunk or stem, from which it can no longer, but with great care, be distinguished by any of its physical qualities.

Something strikingly analogous to this occurs in changes of texture in the mucous membranes of animals when displaced from their normal situation, and subjected to the operation of causes identical with those which act habitually upon the cutaneous covering of the external surface of the body; the mucous membrane thus exposed to the constant impression of the atmosphere, loses much of its natural soft, pulpy, and delicate texture, and assumes an aspect and apparent function greatly analogous to that of the external skin; thus, like the root-bark of vegetables, undergoing a modification of structure no greater than is necessary for its accommodation to the altered location and circumstances it has been accidentally placed in.

It is also well ascertained that the bark of the stems of trees may be made, by placing them under ground, to assume the aspect and function of the root-bark of the same tree—a fact clearly convincive of the great similarity, if not identity of their structure, which alone can explain their mutual convertibility, the one into the other, by mere change of external causes operating upon them.

Similar observations, so far as I am informed, have not been made showing the convertibility of the mucous membrane into skin under peculiar circumstances, into a structure identical with, or greatly similar in structure and function to the cutaneous tissue; but some facts are not wanting to induce a belief of a similar convertibility of mucous into cutaneous tissue when placed in situations favorable to such a result. The retroverted uterus, for example, when placed in permanent contact with the atmosphere, loses its soft and delicate texture and acute sensibility, and becomes cutaneous in its aspect and vital properties. A cutaneous surface similarly changed in its locality, may reasonably be expected to undergo a similar change of aspect and function, as from that of the skin, to the aspect and functions of a mucous tissue.

The mucous membranes are abundantly supplied with bloodvessels and nerves, the latter of which owe their origin partly to the brain, but chiefly to the great sympathetic, or nerve of organic life. The membranes, consequently, whose activity is chiefly devoted to functions appropriated to organic life, have but little sensibility when compared with most other parts of the system, but are largely endowed with the property of irritability, by which their healthy functional activity is almost exclusively subordinated in the economy of animal life.

The distribution of the blood to the surface of the body invested by mucous membrane, is effected almost exclusively through the instrumentality of those very minute bloodvessels which, because of the extreme tenuity of their structure, have been distinguished by the significant appellation of capillary bloodvessels. Few, if any, large arterial or venous trunks are to be found penetrating the substance of these membranes, and hence their comparatively pale complexion, and in some degree their inferior sensibility in their healthy states.

When we consider the structure and normal functions of the mucous membranes in the economy of animal life, it would seem difficult to avoid the conclusion, that they are entitled to receive the most diligent and profound investigation of every medical practitioner. And this estimate of their importance in the animal economy is greatly enhanced, when it is known that one of the most modern and popular theories of fever, makes this greatest scourge of our race to consist in a morbid change in the condition of the mucous surfaces of the gastro-intestinal canal, and that all its usual and more palpable phenomena are but sensible indications of the extent and intensity of these internal morbid changes, which are its proximate though hitherto unknown cause. M. Broussais, who is the illustrious author of this theory of fevers, usually termed idiopathic, but which he has affirmed to be symptomatic only of morbid conditions of the mucous membrane of the gastro-intestinal canal, cannot be charged with having preferred any railing accusation against the verity of anterior systems, but has sustained the positions which he has taken, by evidence drawn from sources, the credit and competency of which it is thought none will be found so illogical or obstinate as entirely to reject, or esteem as of little worth. The Broussain theory of fever which assigns as the proximate cause of this malady an inflamed condition of the mucous membrane of the gastro-intestinal canal, however obnoxious to the objection of being over exclusive in its character, has nevertheless done much towards the establishment of the present improved treatment of febrile diseases.

The comprehension of this theory—of its value and deficiencies—depends essentially upon a thorough acquaintance with the structure, functions, pathological states, and therapeutical relations of the mucous membranes. And should it ever be demonstrated by future research, that the proximate cause of fever consists in obstructed capillary circulation, which is the opinion of the writer of this essay, the investigation of these membranes will fully maintain its relative importance in medical practice, inasmuch as the surfaces under consideration, are abundantly supplied with capillary bloodvessels, and are constantly exposed to the impression of physical and moral causes, peculiarly fitted to derange or obstruct their healthy circulation.

Nashville, May 1, 1845.

